# Functional Anthocyanin-Rich Sausages Diminish Colorectal Cancer in an Animal Model and Reduce Pro-Inflammatory Bacteria in the Intestinal Microbiota

**DOI:** 10.3390/genes9030133

**Published:** 2018-03-01

**Authors:** Javier Fernández, Lorena García, Joaquín Monte, Claudio J. Villar, Felipe Lombó

**Affiliations:** 1 Research Unit “Biotechnology in Nutraceuticals and Bioactive Compounds-BIONUC”, Departamento de Biología Funcional, Área de Microbiología, Universidad de Oviedo. C/Julián Clavería, 7, 33006 Oviedo, Spain; uo186966@uniovi.es (J.F.); cjvg@uniovi.es (C.J.V.); 2 IUOPA (Instituto Universitario de Oncología del Principado de Asturias), C/Fernando Bongera, Edificio Santiago Gascón, 1, 33006 Oviedo, Spain; 3 ISPA (Instituto de Investigación Sanitaria del Principado de Asturias), C/Aldea Cerdeño, 33011 Oviedo, Spain; 4 El Hórreo Healthy Foods SL, Polígono de Granda 17, 33199 Siero, Spain; lorenagarcia@embutidoselhorreo.com (L.G.); joaquinmonte@embutidoselhorreo.com (J.M.)

**Keywords:** flavonoid, anthocyanins, colorectal cancer prevention, *Bilophila wadsworthia*

## Abstract

Colorectal cancer is the fourth most common neoplasia in Europe, where it accounts for 28.2 new cases per 100,000 inhabitants. In an effort to decrease the incidence of this disease, various prevention measures are being studied, one of which are anthocyanin-rich foods. Anthocyanins are potent antioxidant flavonoids mainly found in flowers and colorful fruits and vegetables. These nutraceuticals have diverse biological functions once ingested, including immunomodulatory, anti-inflammatory and antitumor functions. In order to test the preventive effect of these flavonoids against colorectal cancer, an animal model (*Rattus norvegicus* F344) was developed. In this model two doses of azoxymethane (10 mg/kg) and two treatments with dextran sodium sulfate (DSS) were administered to the animals. For 20 weeks they were fed either control rat feed, control sausages, or functional sausages containing 0.1% (*w/w*) of anthocyanins from a mixture of dehydrated blackberries and strawberries. At the end of that period, the animals were sacrificed and their antioxidant plasma levels and digestive tract tissues were analyzed. The results revealed a statistically significant reduction in the number of colon tumors in the functional sausages cohort with respect to the control animals and an increase in the FRAP (ferric reducing ability of plasma) total antioxidant activity in that same cohort. Colon microbiota differences were also examined via metagenomics 16S ribosomal RNA (rRNA) sequencing, revealing a significant reduction in populations of the pro-inflammatory *Bilophila wadsworthia*. Therefore, the design of functional processed meat products, such as ones enriched with anthocyanins, may be an effective strategy for preventing inflammatory digestive diseases and colorectal cancer in human populations.

## 1. Introduction

Colorectal cancer (CRC) is the most frequent type of cancer in the European Union (EU) when considering both sexes combined, with 345,346 new cases and 152,046 deaths in 2012 [1]. Specifically, it is the third most common tumor in men (after lung and prostate cancers) and the second in women (after breast cancer) [2,3,4]. In addition, CRC is increasing every year in Western countries due to environmental risk factors (tobacco, alcohol, chlorine in water) and dietary habits (saturated fat, nitrosamines, benzopyrenes, low consumption of fruit and vegetables), which affect the health of the colon mucosa [5].

Another factor affecting the prevalence of this disease is the timing of its onset. Life expectancy has consistently been on the rise and by 2025 about 10.4% of the world population will be over 65 years old [6,7]. Since CRC progression requires diverse genetic mutations, it usually appears later in life, resulting in an increased incidence in the modern population.

The initial stages of tumor generation in the colon mucosa, as shown by animal and human studies, take place in the crypt cells, which are present in large quantities in this epithelium. These cells are responsible for carrying out the processes of continuous cell multiplication necessary to maintain the epithelium’s structure and function [8]. Mutations occurring as part of the cellular division of one of these stem cells at the bottom of a crypt will generate a cell with uncontrolled growth, eventually resulting in a crypt with aberrant morphology (aberrant crypt foci, ACF). Later these will develop into a mucosal microadenoma, which will then evolve towards a large adenoma (polyp) and, finally, into an adenocarcinoma with metastatic capabilities [9,10].

The described tumorigenic process arising from the initial ACF in colon mucosa can be altered or even stopped by the presence of some nutraceutical compounds in the colon lumen, such as polyphenols. Polyphenols are plant metabolites with many important health effects in humans once ingested (cardioprotective, antioxidant, antitumor, etc.) [11,12]. Anthocyanins are one family of these polyphenols. Anthocyanins are responsible for the intense colors of blue and red pigmented flowers and fruits, where they usually are present as glycosylated anthocyanins [13,14,15,16,17]. Once ingested, anthocyanins are deglycosylated by intestinal glucosidases and their aglycons (anthocyanidins) are absorbed through the intestinal mucosa, exerting their biological effect on various tissues [11,18].

Similar to other polyphenols, the biological effects of ingested anthocyanins include anti-inflammatory [19,20,21,22] and antitumor activities against human cancer cell lines of various origins (such as breast, colon, glioblastoma, liver, lung, melanoma, ovary or prostate) [23,24,25,26,27]. These antitumor effects seem to be related to the inhibition of cancer cell proliferation by increasing oxidative stress biomarkers and by inducing apoptosis through the mitochondrial pathway. They also appear to affect cancer cell migration and invasion by inhibiting matrix metalloprotease expression, such as MMP-2 and MMP-9, and by inhibiting epithelial–mesenchymal transition. These anthocyanins are also able to protect the genome of epithelial cells from factors that may induce mutations and promote the inhibition of pro-inflammatory pathways, such as those dependent on COX-2 and NF-κB [28,29,30,31,32,33,34,35,36]. All these activities are of interest regarding CRC prevention.

Based on this knowledge, the present study focused on analyzing the potential preventive effects of functional processed red meat products, sausages, containing 0.11% anthocyanins from a 1:1 mixture of dehydrated blackberries and strawberries against CRC in an animal model (*Rattus norvegicus* F344). This matrix was specifically selected because processed meat products lack preventive effects against CRC and, in some cases, are even a risk factor for the disease due to their high content of saturated fat, heme groups and, in some cases, nitrosamines [37,38,39,40]. In this study, these tumors were chemically induced using a mixed treatment of azoxymethane (AOM) and dextran sodium sulfate (DSS). Multiple parameters were analyzed in these experimental animals, such as body weight, caecum weight, number of hyperplastic Peyer’s patches, total antioxidant activity in blood plasma, number of colon polyps and the tumor mucosa surface area. The intestinal microbiota was also examined in the three cohorts (rat feed, control sausages, anthocyanins functional sausages), revealing significant differences between them.

## 2. Materials and Methods

### 2.1. Animals and Experimental Design

A total of 30 male Fischer 344 rats were maintained in the Animal Facilities at the University of Oviedo (authorized facility No. ES330440003591). All rat experiments were approved by the Ethics Committee of the Principality of Asturias (authorization code PROAE 23/2016, April 2016).

The rats (five weeks old) were divided into three cohorts of 10 individuals each and fed ad libitum. Cohort 1 was fed universal feed (2014 Teklad Global 14% Protein Rodent Maintenance Harlan diet feed, Harlan Laboratories, Barcelona, Spain). This feed contained 14.3% protein, 4% fat, 48% carbohydrates, 22.1% fiber, 4.7% ashes.

Cohort 2 was fed 10 g/day/rat of feed and 20 g/day/rat of control sausages (1 kg of porcine loin ribbon, 25 g NaCl, 15 g dehydrated garlic, 3 g dry black pepper). The loin ribbon composition was 30% protein, 8% lipids, 70% humidity, 0.1% ashes. The dehydrated garlic contained 1.3% of ether-extractable compounds and the dry black pepper contained 2% of essential oil and 7% of ashes.

Cohort 3 was fed 10 g/day/rat of feed and 20 g/day/rat of functional sausages containing anthocyanins (same formula per kg, but with 100 g of 1:1 mixed dehydrated strawberry and blackberry powder). The 1:1 dehydrated berry powder contained 1.1% anthocyanins (59% of cyanidin-3-glucosides and 41% of pelargonidin-3-glucosides). Therefore, the functional sausages contained 1.1 g of anthocyanins per kg (i.e., 0.11% anthocyanins).

### 2.2. Colorectal Cancer Induction and Monitoring

One week after the animals arrived at the facility, the three corresponding diets were started. After one week of eating the corresponding diet, CRC was induced in eight rats from each cohort. The two other rats were kept free of CRC induction as absolute control animals. CRC induction was carried out in those eight rats of each cohort using Azoxymethane (AOM, Sigma-Aldrich, Madrid, Spain) dissolved in sterile saline (0.9% NaCl) at a concentration of 2 mg/mL. This AOM solution was injected intraperitoneally at a final concentration of 10 mg per kg body weight. The AOM treatment was repeated seven days after the first injection (weeks 2 and 3). The absolute control animals received sterile saline in both injections.

In week 4 and 15, rats received drinking water for seven days containing 3% and 2% dextran sodium sulfate (DSS, 40.000 g/Mol, VWR), respectively. This ulcerative colitis step was repeated twice because it enhances the pro-carcinogenic effect caused by AOM administration.

The rats were sacrificed by pneumothorax 20 weeks after the first administration of AOM.

Throughout the entire process the rats were monitored for body weight and stool consistency/rectal bleeding.

### 2.3. Physical Measures

The rats were weighted regularly during the 20 experimental weeks: at reception of the animals (week 1), at each of the AOM administrations (weeks 2 and 3), and at weeks 4, 7, 15 and 20.

### 2.4. Blood and Tissue Samples

The rats were anesthetized (isoflurane) and sacrificed (pneumothorax) at week 20. This allowed for the extraction of 2 mL of blood from the heart, which was then centrifuged at 3.000 rpm for 15 min, and then the plasma was frozen.

The small intestine was removed fresh and the hyperplastic Peyer’s patches were counted. Their number in the experimental animals was calculated and compared with respect to the small intestine Peyer patches of the two absolute control animals from each cohort (animals 9 and 10).

The caecums were weighed immediately after sacrifice using a precision scale and then frozen at −20 °C.

Finally, the colon was opened longitudinally and washed with PBS (phosphate buffer saline) before keeping it in 4% formaldehyde at 4 °C. Fixed colons were meticulously examined with a precision gauge in order to count the number of tumors larger than 1 mm on the inner mucosa surface. The biggest detected tumors were 11 mm in diameter. The shape of the tumors was identified as pedunculated (a disc connected via a peduncle to the colon mucosa), plane irregular, plane circular and spherical. Finally, the total tumor-affected area was calculated.

### 2.5. Total Antioxidant Capacity in Blood Plasma

Total antioxidant activity was measured in plasma samples using a commercial FRAP (ferric reducing activity of plasma) assay kit (Ref. Kf-01-003, Bioquochem SL, Llanera, Spain). A standard curve of different Trolox (a vitamin E analogue) concentrations was used for comparison.

### 2.6. Genomic DNA Extraction and 16S Ribosomal RNA Sequencing for Metagenomics

Genomic DNA (gDNA) was extracted from 200 mg of frozen caecum feces using E.Z.N.A.^®^ DNA Stool Kit (Ref. D4015-02, VWR, Madrid, Spain) and provided 200 µL of genomic DNA. These gDNA samples were then quantified using a BioPhotometer^®^ (Eppendorf, Madrid, Spain) and their concentrations diluted to 6 ng/µL. The diluted samples were used for performing polymerase chain reaction (PCR) amplification, following the protocol of the Ion 16^TM^ Metagenomics kit (Thermo Fischer Scientific, Madrid, Spain).

PCR amplification products were utilized to create a library using the Ion Plus Fragment Library kit for AB Library Builder^TM^ System (Cat. No. 4477597, Thermo Fischer Scientific), with sample indexing using the Ion Xpress^TM^ Barcode Adapters 1-96 kit (Cat. No. 4474517, Thermo Fischer Scientific). Template preparation was performed using the ION OneTouch^TM^ 2 System and the ION PGM^TM^ Hi-Q^TM^ OT2 kit (Cat. No. A27739, Thermo Fischer Scientific). Metagenomics sequencing was performed using ION PGM^TM^ Hi-Q^TM^ Sequencing kit (Cat. No. A25592, Thermo Fischer Scientific) on the ION PGM^TM^ System. The chips used were the ION 314^TM^ v2, 316^TM^ v2 or 318^TM^ v2 Chips (Cat. No. 4482261, 4483188, 4484355, Thermo Fischer Scientific) with various barcoded samples per chip.

### 2.7. Phylogenetic Analysis

The consensus spreadsheet for each metagenomics sequencing was downloaded from ION Reporter software (version 5.6, Life Technologies Holdings Pte Ltd, Singapore). This spreadsheet includes the percentages for each taxonomic level and was used for comparing frequencies between individual rats and cohorts.

### 2.8. Statistical Methods

The normality of the different variables was tested using Shapiro–Wilk’s test. In light of these results, the data were then expressed as the mean value ± standard error of mean (S.E.M.) and parametric methods were used for statistical analyses. The equality of variances was tested using Levene’s test and then the differences among cohorts were tested by a one-way ANOVA (analysis of variance). When the quantitative data was not normal, the non-parametric Mann–Whitney U test was used.

The graphic representation of all the data was carried out using GraphPad Prism software, (version 7, GraphPad Software, San Diego, CA, USA). In each case, a *p* value < 0.05 was considered statistically significant (* *p* < 0.05; ** *p* < 0.005; *** *p* < 0.0005; **** *p* < 0.0001).

## 3. Results

### 3.1. Effect of Anthocyanin-Rich Functional Sausages on Body Weight

**Figure 1:**
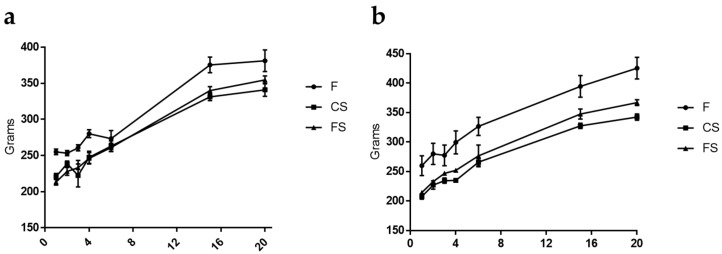
(**a**) Evolution of body weight throughout the whole experiment for the eight rats with colorectal cancer (CRC) induction in the three cohorts. Body weight was measured at weeks 1, 2, 3, 4, 7, 15 and 20. When the animals were sacrificed the mean value for the feed cohort was 311.2 ± 39.6 g, for the control sausages cohort was 341.0 ± 25.6 g and for the functional sausages cohort was 354.7 ± 15.1 g; (**b**) Evolution of body weight along the whole experiment for the two absolute control rats (no CRC induction) in the three cohorts. Body weight was measured at weeks 1, 2, 3, 4, 7, 15 and 20. When the animals were sacrificed the mean value for the feed cohort was 425.5 ± 26.1 g, for the control sausages cohort was 342.5 ± 7.7 g and for the functional sausages cohort was 367.0 ± 7.0 g. F: feed cohort, CS: control sausage cohort, FS: functional sausage cohort.

Animals from the three cohorts maintained a constant weight gain throughout the 20 experimental weeks (the first AOM challenge for CRC induction took place at week 3). However, it is evident from Figure 1a that during both DSS events (weeks 4 and 15), the animals of the feed cohort under CRC induction suffered a slowdown in weight gain. Also, rat number 3 from the feed cohort died during the second DSS challenge as a consequence of intense rectal bleeding caused by this transitional ulcerative colitis episode, which was a necessary pro-inflammatory step in order to increase the final numbers (and volume) of the AOM-generated tumors.

In the absolute control animals (rats 9 and 10 in each cohort), the body weight gain was more constant throughout the whole experiment, as these animals were not getting AOM or DSS.

### 3.2. Effect of Anthocyanin-Rich Functional Sausages on Hyperplastic Peyer Patches

**Figure 2:**
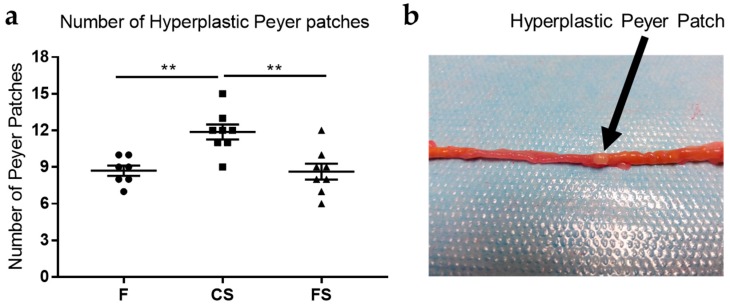
(**a**) Mean number of hyperplastic Peyer patches in the small intestines from each cohort (rats 1 to 8 in the three groups). In comparison, absolute control animals (those lacking azoxymethane (AOM) and dextran sodium sulfate (DSS) treatments) from each cohort showed a mean value of 5.83 ± 0.16 hyperplastic Peyer patches. Although in the control sausage cohort the average value increased (11.8 ± 0.6 patches) with respect to the feed cohort (8.7 ± 0.4 patches) and this difference was statistically significant (*p* value 0.0032), the number decreased close to that of the feed cohort value in the case of the functional sausage cohort (8.6 ± 0.6 patches). The difference between both sausage cohorts was also statistically significant (*p* value 0.0018); (**b**) Picture showing a representative hyperplastic Peyer patch in the small intestine mucosa (black arrows), which appears as a rounded protruding white 2–3 mm oval. F: feed cohort, CS: control sausage cohort, FS: functional sausage cohort.

The number of hyperplastic Peyer patches in the small intestine was quantified when the animals were sacrificed. Peyer patches contain high amounts of lymphocytes and are located in the mucosa layer of the small intestine. These lymphoid nodules can become hyperplastic and are therefore easily visible in the small intestine as rounded, protruding, white 2–3 mm ovals (Figure 2).

In this study, differences in the Peyer patch mean values were statistically significant between the control sausages and feed cohorts, and also between the control sausage and functional sausage cohorts (Figure 2).

### 3.3. Effect of Anthocyanin-Rich Functional Sausages on Blood Total Antioxidant Capacity

**Figure 3:**
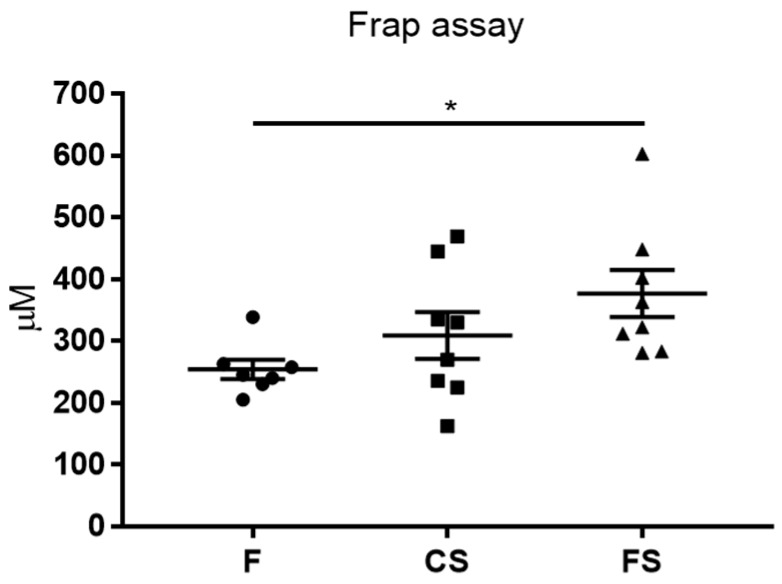
Mean numbers of blood total antioxidant capacity, in µM Trolox equivalents, from each cohort (only rats 1 to 8 in the three groups). The only statistically significant difference is found between the functional sausage cohort (376.8 ± 38.3) and the feed cohort (254.2 ± 15.7), with a *p* value of 0.048. This value was slightly higher in the control sausage cohort (309 ± 37.9), but this difference was not statistically significant. F: feed cohort, CS: control sausage cohort, FS: functional sausage cohort.

The rats in the functional sausage cohort showed a much higher total antioxidant capacity in the blood plasma (359.8 µM Trolox equivalent) than those in the feed cohort (252.9 µM Trolox equivalent), and this difference was statistically significant (Figure 3). The rats in the control sausage cohort showed an increase in the antioxidant capacity with respect to the feed cohort, but this increase was not statistically significant (Figure 3).

### 3.4. Effect of Anthocyanin-Rich Functional Sausages on Caecum Weight

**Figure 4:**
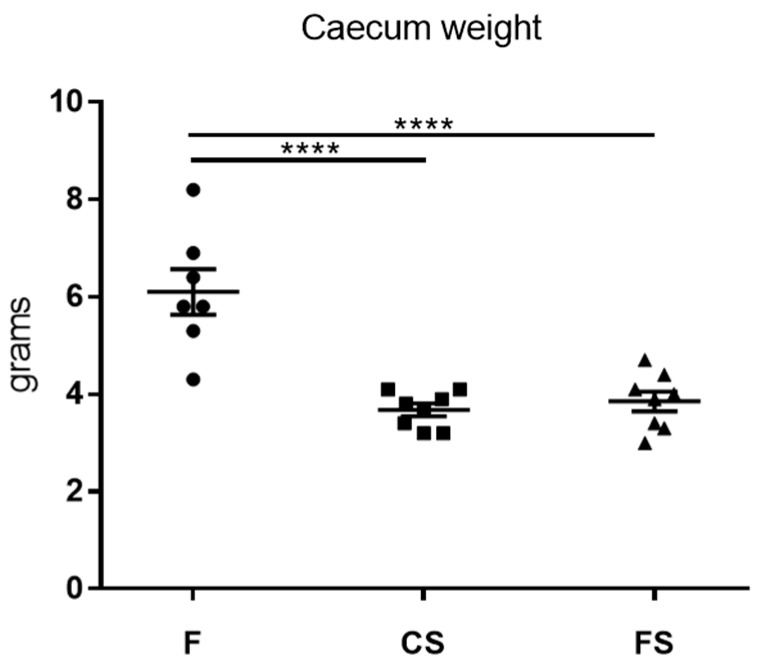
Mean numbers of caecum weight in grams for each cohort (only rats 1 to 8 in each group). These mean values were decreased in the control sausage (3.67 ± 0.1 g) and functional sausage (3.85 ± 0.2 g) cohorts with respect to the feed cohort (6.1 ± 0.4 g) and these differences were statistically significant (*p* values 0.0001). F: feed cohort, CS: control sausage cohort, FS: functional sausage cohort.

Statistically significant differences in the caecum weight values were observed between the feed cohort and the control sausage cohort, as well as between the feed cohort and functional sausage cohort (Figure 4).

### 3.5. Effect of Anthocyanin-Rich Functional Sausages on Number of Polyps and Tumor-Affected Area

**Figure 5:**
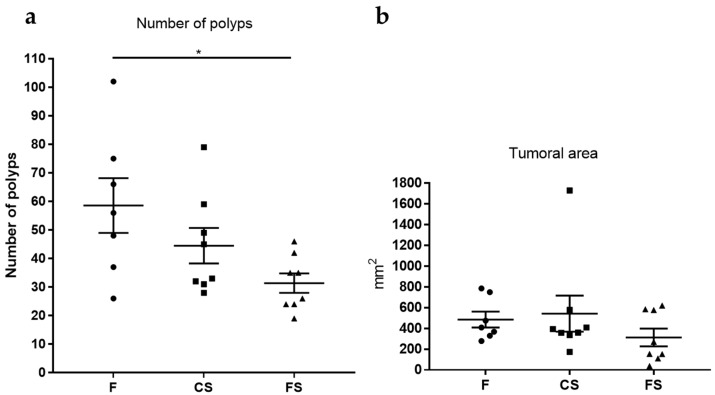
(**a**) Average number of colon polyps from each cohort (rats 1 to 8 in the three groups). The average number of polyps decreased in the case of the functional sausage (31.38 ± 3.3) cohort with respect to the feed cohort (58.5 ± 9.5) and this difference was statistically significant (*p* value 0.0241). However, no statistically significant difference was observed in the case of the control sausage cohort (44.5 ± 6.2). The absolute control rats showed zero colon polyps (rats 9 and 10 from each cohort); (**b**) For each animal, the area of each single polyp was calculated, and then these values were added. This figure shows the average value in mm^2^ of the sum of polyp areas from all polyps in all animals from each cohort. Although a reduction was observed in the mean affected area of the functional sausage cohort (313.1 ± 85.3 mm^2^) with respect to the feed cohort (484.8 ± 76.6 mm^2^) and the control sausage cohort (541.8 ± 174.1 mm^2^), this reduction was not statistically significant. F: feed cohort, CS: control sausage cohort, FS: functional sausage cohort.

The colon mucosa were analyzed for the number of polyps. In these experiments, the polyp size ranged from 1 mm to 11 mm diameter. A statistically significant difference was only observed among rats in the feed cohort and those in the functional sausage cohort, which showed a drastic 46.4% reduction in the number of polyps (Figure 5). The number of polyps was also reduced in the case of the control sausage cohort with respect to the feed cohort, but this reduction was not statistically significant (Figure 5).

The area of each polyp present in a given colon mucosa was calculated according to its shape and the total tumor area was computed for each animal. The functional sausage cohort showed a 35.4% reduction in this parameter with respect to the feed cohort and a 42.2% reduction with respect to the control sausage cohort. However, these reductions were not statistically significant (Figure 5).

### 3.6. Effect of Anthocyanin-Rich Functional Sausages on Intestinal Microbiota

The intestinal metagenomics sequencing of the three cohorts showed considerable differences in average phyla compositions when comparing the feed cohort and the other two (control sausage and functional sausage) (Table 1).

The main difference between these cohorts is the near absence of *Proteobacteria* in the rats of the feed cohort (0.32%), whereas *Proteobacteria* is the second most common phylum in the control sausage cohort (15.8%) and the third most common phylum in the functional sausage cohort (10.4%) (Table 1).

**Figure 6:**
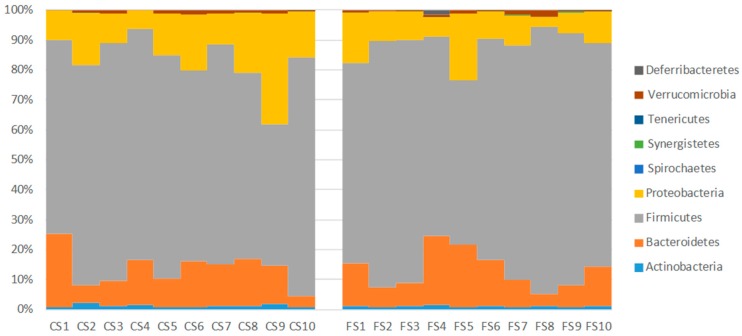
Intestinal microbiota composition, at phylum level, in the rats belonging to the control sausage cohort (CS1 to CS10), and in the rats belonging to the functional sausage cohort (FS1 to FS10). CS: control sausage cohort, FS: functional sausage cohort.

Also, greater biodiversity was found in the two sausage cohorts, as evidenced by the number of phyla (although in low proportions) present in their composition (Table 1). When analyzing the individual proportions of the phyla in these 20 rats, a homogeneous distribution between all rats in each cohort is found (Figure 6), with the predominant phylum being Firmicutes (82.9% in the feed cohort, 69.6% in the control sausage cohort, and 75.1% in the functional sausage cohort) (Table 1).

In the case of the rats belonging to the control sausage cohort, the second most abundant phylum is Proteobacteria, but in the case of the functional sausage cohort, the second most abundant phylum is Bacteroidetes (12.1%, with 12.4% in the control sausage cohort) (Figure 6).

**Figure 7:**
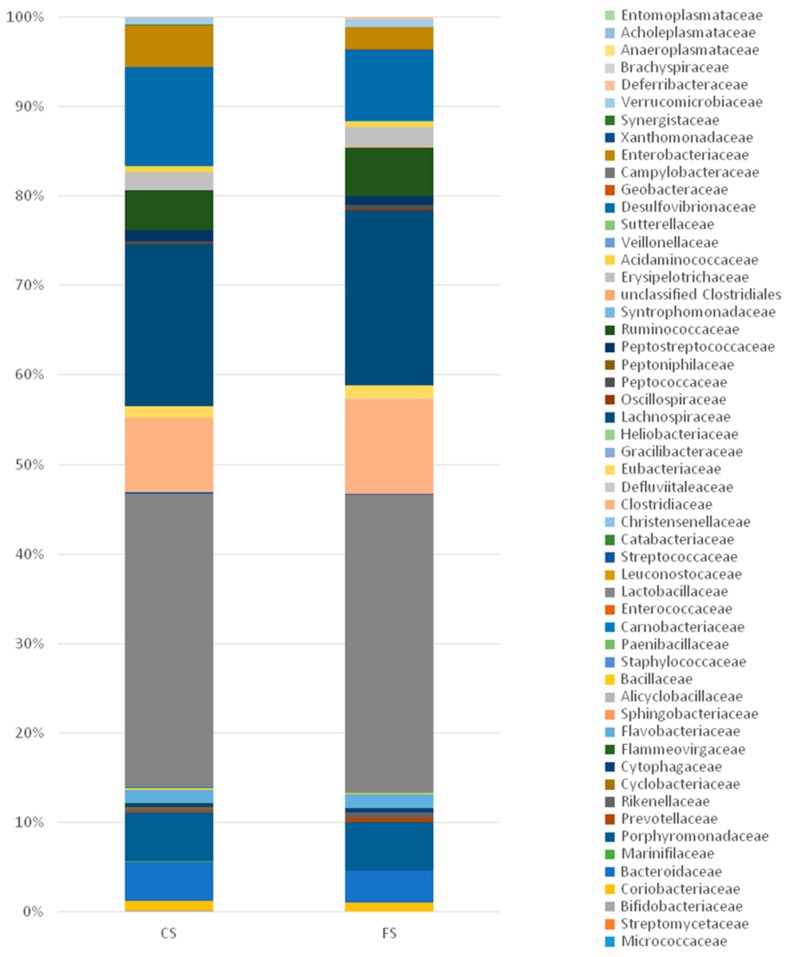
Differences in average intestinal microbiota composition at the family level for the rats belonging to both sausage cohorts. CS: control sausage cohort, FS: functional sausage cohort.

**Figure 8:**
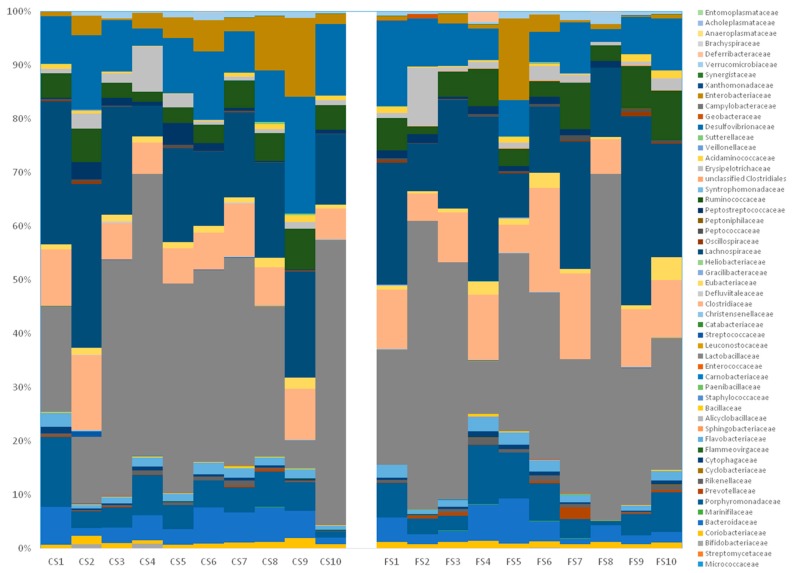
Intestinal microbiota composition at the family level in the rats belonging to the control sausages cohort (CS1 to CS10), and in the rats belonging to the functional sausages cohort (FS1 to FS10). CS: control sausage cohort, FS: functional sausage cohort.

At the family level, the two cohorts fed with meat products showed similar compositions (Figure 7 and Figure 8), but with clear increases in distinct families with respect to the feed cohort. For example, Lactobacillaceae is more abundant in both the control and the functional sausage cohorts (32.80% and 33.23%, versus 7.72% in the feed cohort), as well as Desulfovibrionaceae (11.09% and 7.96% in the sausage cohorts, versus 0.26% in the feed cohort), Bacteroidaceae (4.44% and 3.51%, respectively, versus 1.93% in the feed cohort), Enterobacteriaceae (4.65% and 2.38%, respectively, versus 0% in the feed cohort), and Coriobacteriaceae (1.04% and 1.04%, respectively, versus 0% in the feed cohort) (Figure 7).

In both sausage cohorts, the main differences at the family level are observed with respect to three families (Figure 7). Clostridiaceae is more abundant in the functional sausage cohort (10.56%) with respect to the control sausages cohort (8.28%). Desulfovibrionaceae and Enterobacteriaceae are less present in the functional sausages cohort (7.96% and 2.38%, respectively) with respect to the control sausages cohort (11.09% and 4.65%, respectively).

**Figure 9:**
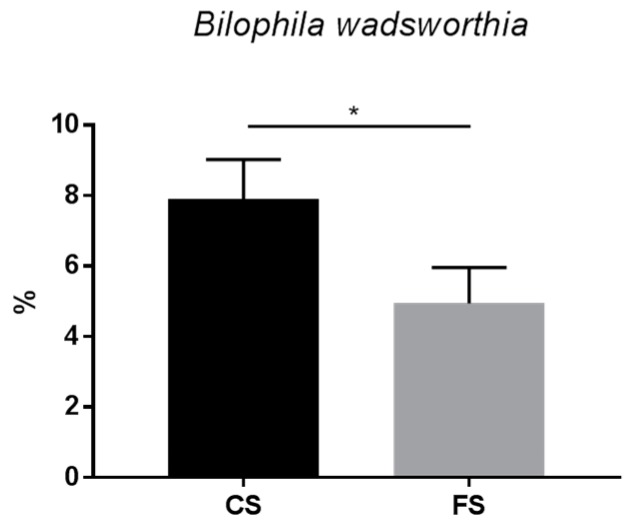
Differences in intestinal microbiota composition at the genus and species level for the rats belonging to the control sausage cohort and the functional sausage cohort, which are only found for *Bilophila wadsworthia* (*p*-value 0.1).

At the genus level, the only significant difference was found in the Desulfovibrionaceae family with respect to the species *Bilophila wadsworthia*, which represents 7.90% of all metagenomics in the control sausage cohort, but only 4.94% in the rats of the functional sausage cohort (Figure 9).

## 4. Discussion

In this study, the sausage composition was designed to have a low saturated fat content by using porcine loin ribbon as the main ingredient (30% protein and only 8% lipids). Garlic powder and black pepper powder were also added to this formula. This composition was used for the control sausages (see Materials and Methods, Section 2). For the functional sausage version, a dehydrated strawberry and blackberry powder mix, a rich source of anthocyanins (1.1% in this case), was added for a final composition of 0.11% anthocyanins, 0.15% garlic and 0.03% black pepper.

The three cohorts of rats were fed a diet of either feed, control sausages or functional sausages for a total of 20 weeks, and colon tumors were induced chemically with AOM and enhanced with DSS. One of the animals in the feed cohort died during the second DSS challenge due to intense rectal bleeding caused by this transient ulcerative colitis stage. The deleterious effect of AOM injections (two, in weeks 2 and 3) and DSS challenges (two, in weeks 4 and 15) was also observed when analyzing the weight gain of the rats throughout the experiment. In Figure 1, considerable decelerations in weight gain at those points in the experiment can be observed, especially in the rats from the feed and control sausage cohorts. A possible explanation for this apparent protection against AOM and/or DSS inflammation in the case of the functional sausage cohort may be the known anti-inflammatory effect of anthocyanins [19,20,21,22,41].

At the end of the experiment, in the 20th week, all 29 surviving animals were sacrificed. In order to assess the effect of the diets on CRC, certain histological parameters were analyzed: number of hyperplastic Peyer patches, blood total antioxidant capacity, caecum weight, number of colon tumors, and total tumor area in the colon mucosa (Figure 2, Figure 3, Figure 4 and Figure 5).

In the case of hyperplastic Peyer patches, statistically significant differences were observed between the functional sausage cohort and the control sausage cohort (Figure 2). These small intestinal mucosa structures are abundant in lymphocytes and become hyperplastic when they react to alterations in the digestive tract, which affects the animal’s immune condition, as may happen in response to some toxins [42]. In the present study, the magnitude of hyperplastic Peyer patches was used as a measurement of the general pro-inflammatory condition of the animals in response to the CRC induction treatment. The results revealed lower numbers of hyperplastic Peyer’s patches in the functional sausage cohorts. This can be explained by the anti-inflammatory effect of the anthocyanins in the functional sausages (0.11% total content).

Another possible beneficial effect of anthocyanins on the functional sausage cohort was demonstrated when analyzing the plasma total antioxidant capacity (FRAP assay, Figure 3). Specifically, a statistically significant difference was found with respect to the feed cohort animals. These results suggest that these flavonoids, similar to the other members of this large family of plant nutraceuticals, are potent antioxidants and that this activity may help protect against DNA damage and cancer progression [28,43,44].

The final histological values measured were the number of colon tumors and the total tumor area in the colon mucosa (Figure 5). In terms of the number of colon tumors, there was a statistically significant reduction in the functional sausage cohort (31.38 ± 3.3) with respect to the feed cohort (58.5 ± 9.5). This reduction was also evident with respect to the control sausage cohort (44.5 ± 6.2), though lacking statistical significance. From these tumor numbers it is clear that the addition of anthocyanins to the functional sausages formula provided protection against CRC in this animal model. Thus, the results demonstrate that this formula produces a functional processed red meat product with preventive properties against CRC.

The control sausage cohort showed higher numbers of colon tumors than the functional sausage cohort, as expected in the experimental design, but surprisingly this control meat cohort displayed fewer tumors than the animals of the feed cohort (although lacking statistical significance), which were fed the optimized vegetable diet for this species. One possible explanation is that the control (and the functional) sausage formula was designed to include only 8% lipids (mainly saturated fat) and no nitrates or nitrites (known precursors of nitrosamines). Instead, 0.15% garlic powder and 0.03% black pepper powder were used in the sausages as antimicrobial additives. Both of these spices have known antitumor properties with respect to CRC, due to the allicin content of garlic and the piperine content of black pepper [45,46]. These nutraceuticals may be responsible for the observed reduction in the number of colon tumors in the control sausages cohort with respect to the feed cohort. Regardless, the addition of 0.11% anthocyanins to this base formula contributed to the design of a functional processed meat with great antitumor prevention properties (Figure 5).

Once the measurement of the histological values was completed, the intestinal microbiota composition was determined through metagenomics 16S ribosomal RNA (rRNA) sequencing of cecal content. At the phylum level, prominent differences between the feed cohort and the two types of meat cohorts were observed (Table 1 and Figure 6). However, the corresponding phylum compositions of the 10 animals in each meat cohort are generally quite similar (Figure 6). Both sausages cohorts contain high proportions of Proteobacteria (15.8% in the control sausages cohort, 10.4% in the functional sausages cohort). Thus it can be deduced the addition of meat to the diet allows for a substantial increase in Proteobacteria titers (Table 1). This is in line with studies using animal models and human populations, where the presence of higher quantities of meat products in the diet is associated with a dysbiosis towards Proteobacteria populations in intestinal microbiota and with a pro-inflammatory state [47,48]. This pro-inflammatory state related to a greater ingestion of meat products has been associated with increased incidence of inflammatory bowel disease, Crohn disease and CRC [47,48]. By contrast, in the feed cohort, the high percentage of fiber enhances higher populations of Firmicutes (82.99%) and Bacteroidetes (16.42%) (Table 1).

At a lower taxonomic level, the family composition is quite similar for both meat product cohorts, although three pronounced differences can be observed (Figure 7 and Figure 8) with respect to Clostridiaceae (more prevalent in the functional sausage cohort), Enterobacteriaceae (reduced in the functional sausage group) and Desulfovibrionaceae (also reduced in the functional sausage rats) (Figure 7). This reduction in the Desulfovibrionaceae, from 11.09% in the control sausage animals to 7.96% in the functional sausage cohort, is worth noting, as this is the only family where the metagenomics data shows a statistically significant difference at the species level as well. This difference was detected in the case of *Bilophila wadsworthia*, an intestinal sulphite-reducing bacteria which generates H_2_S (Figure 9). *B. wadsworthia* increases in meat-based diets, a type of diet which enhances bile acids secretion due to its higher proportion of fat content. This higher intestinal concentration of bile acids is able to enhance intestinal populations of *B. wadsworthia*, which are highly resistant to bile acids. The abundance of this species in persons affected by intestinal diseases (appendicitis, inflammatory bowel disease, etc.) has been associated with the development of pro-inflammatory intestinal disorders and, eventually, CRC, as a result of high intestinal genotoxic H_2_S levels [49,50,51,52,53]. This pro-inflammatory effect for *B. wadsworthia* has been recently demonstrated in a pathogen-free mouse model, where oral administration of this bacterium induced an inflammatory status (reduction of body weight, hepatosplenomegaly, elevated serum IL-6 and amyloid A protein) [54].

Based on this data, it can be noted that the addition of anthocyanins to a functional sausage formula was able to generate diverse intestinal microbiota changes, mainly in three taxonomic families (Clostridiaceae, Enterobacteriaceae and Desulfovibrionaceae) and, in the case of Desulfovibrionaceae, these changes were mainly due to a substantial reduction in *B. wadsworthia* populations. This species is associated with meat-based diets in animals and humans and seems to trigger pro-inflammatory intestinal diseases (or at least is not present in healthy individuals). Also, this bacterium generates high rates of the genotoxic compound H_2_S. Its reduced populations in the case of the functional sausage cohort may be a protective side effect of anthocyanin ingestion. However, the potential specific effect of anthocyanins on *B. wadsworthia* growth has not yet been analyzed.

With respect to CRC prevention, purified anthocyanin extracts from plants such as blue maize or blue sweet potato have shown antitumor effects in in vitro against cancer cell lines [25]. For example, in vitro experiments with the Caco-2 colon cancer cell line showed a 40% reduction in survival after treatment with extracted anthocyanins from blue potatoes [55]. Similar effects have been observed in other colon cancer cell lines such as HT-29 [56,57]. These flavonoids have also shown protective effects against CRC in in vivo animal models. For example, in an *Apc* gene mice model, colon tumor numbers were reduced to one third by diet supplementation with a 0.12% anthocyanins extract or 10% blue sweet potato (which is equal to 0.12% anthocyanins total content in the diet) [58]. A similar reduction in colon tumors has also been observed in mouse models using other types of anthocyanins, such as a bilberry extract [59].

Anthocyanins show important anti-inflammatory properties. For example, these flavonoids are able to reduce CD3^+^ T lymphocytes in gut mucosa, an important cell lineage associated to inflammation cascades [60]. However, anthocyanins have been described as important modulators of gut microbiota as well. For example, anthocyanin supplementation in animal models for CRC have been able to reduce populations of pathogenic bacteria (such as *Enterococcus* sp. and *Desulfovibrio* sp.), and at the same time to increase *Eubacterium rectale*, *Faecalibacterium prausnitzii* and other probiotic species populations in gut [61]. This gut microbiota modulation can explain the important differences described in this study between animals receiving both types of sausage diet. All these glycosylated flavonoids suffer the enzymatic hydrolysis of their sugars once they enter the intestinal environment, giving rise to anthocyanidins aglycons. Anthocyanidins are the molecules finally absorbed by intestinal cells and therefore the active derivatives in this family of flavonoids [11,18].

The CRC prevention in this study is most probably due to anti-inflammatory effects of anthocyanins supplementation, including an eventual downregulation of inflammatory pathways as it has described for these flavonoids, but also the reduction of important pro-inflammatory gut bacterial populations, such as *B. wadsworthia*. Another important protective effect in this CRC study is the increased antioxidant status also showed in the animals receiving the functional sausage diet. In conclusion, this study has formulated a functional processed meat product (anthocyanin-rich sausages) with CRC prevention properties that may be a possible alternative to diet interventions in order to promote healthier human populations, especially in countries with a high incidence of intestinal dysbiosis caused by an excess of dietary meat products.

## Figures and Tables

**Table 1:** Average percentage composition of intestinal microbiota at phylum level for the three cohorts studied.

	Feed (%)	Control Sausage (%)	Functional Sausage (%)
Actinobacteria	0.06	1.22	1.08
Bacteroidetes	16.42	12.44	12.11
Firmicutes	83.00	69.61	75.18
Proteobacteria	0.32	15.84	10.49
Spirochaetes	0.00	0.01	0.02
Synergistetes	0.00	0.02	0.06
Tenericutes	0.18	0.00	0.01
Verrucomicrobia	0.01	0.85	0.87
Deferribacteretes	0.00	0.02	0.19
